# Influence of uncontrolled diabetes mellitus on periodontal tissues during orthodontic tooth movement: a systematic review of animal studies

**DOI:** 10.1186/s40510-017-0159-z

**Published:** 2017-02-06

**Authors:** Shariq Najeeb, Fahad Siddiqui, Saad Bin Qasim, Zohaib Khurshid, Sana Zohaib, Muhammad Sohail Zafar

**Affiliations:** 1 Riyadh Consultative Clinics, Imam Saud Bin Abdul Aziz Road, P.O. Box 361724, Riyadh, Al Murooj 11313 Saudi Arabia; 2 Department of Pediatric Dentistry, Rutgers School of Dental Medicine, New Brunswick, NJ USA; 3 Materials Science and Engineering Department, Kroto Research Institute, University of Sheffield, Sheffield, UK; 4 Department of Prosthodontics and Implantology, King Faisal University, Al-Hofuf, Saudi Arabia; 5 School of Biomedical Engineering, King Faisal University, Al-Hofuf, Saudi Arabia; 6 Department of Restorative Dentistry, Taibah University, Madinah Al Munawwarah, Saudi Arabia

## Abstract

Diabetes mellitus (DM) may adversely affect periodontal tissues during orthodontic tooth movement (OTM). The aim of this review is to systematically analyze and review animal studies investigating the effect of DM on periodontal tissues during OTM. An electronic search was conducted via PubMed/Medline, Google Scholar, Embase, ISI Web of Knowledge, and Cochrane Central Register of Controlled Trials (CONTROL) using the keywords “diabetes,” “orthodontics,” and “tooth movement” for studies published between January 2000 and August 2016. After elimination of duplicate items, the primary search resulted in 89 articles. After exclusion of irrelevant articles on the basis of abstract and title, full texts of 25 articles were read to exclude additional irrelevant studies. Seven animal studies were included in this review for qualitative analysis. When compared to healthy animals, more bone resorption and diminished bone remodeling were observed in diabetic animals in all studies. Furthermore, DM decreased the rate of OTM in one study, but in another study, DM accelerated OTM. DM may adversely affect bone remodeling and tooth movement during application of orthodontic forces. However, a number of potential sources of bias and deficiencies in methodology are present in studies investigating the association between OTM and DM. Hence, more long-term and well-designed studies are required before the exact mechanism and impact of DM on outcomes of orthodontic treatment is understood.

## Review

### Introduction

Diabetes mellitus (DM) is a chronic disease which is characterized by an impaired production or utilization of insulin, leading to high amounts of blood glucose. High amounts of glucose may damage the blood vessels, nerves, and body organs. There are two main types of DM. Type 1 DM (T1DM) is caused by immune-mediated destruction of beta cells of the pancreas which leads to insufficient production of insulin. Type 2 DM (T2DM) occurs when the body is nonresponsive to insulin. In later stages of T2DM, a diminished production of insulin may also develop. Uncontrolled DM may lead to a variety of complications including delayed wound healing, stroke, renal failure, anxiety, retinopathy, and limb amputation. In 2013, it was estimated that there are approximately 382 million diabetic individuals worldwide, and the number is expected to rise to 592 million by 2035 [1]. Due to an impaired immune system and a reduced salivary flow, individuals with DM have a higher incidence of dental caries, periodontal disease, and oral infections [2–5]. Additionally, uncontrolled juvenile DM mellitus (JDM) has been observed to retard growth in children [6, 7].

Orthodontics involves inducing tooth movement by removable and fixed appliances with or without modification of craniofacial growth in order to treat malalignment of the teeth and/or jaws. A number of systemic factors have been observed to adversely affect orthodontic treatment. Animal studies have revealed hypercalciuria and diminished intestinal absorption of calcium in diabetic subjects, indicating increased demineralization of the bone [8, 9]. Similarly, as observed in clinical studies, DM reduces osteoblastic function which results in decreased bone density [10]. Also, it has been suggested that DM alters mandibular growth and craniofacial development [11]. Indeed, clinical studies have also shown that diabetes induces an increased production of pro-inflammatory factors which accelerate bone resorption, leading to a reduced bone mineral density [12]. Patients with DM have been observed to have increased prevalence and intensity of periodontal disease when compared to systematically healthy patients. Prolonged increased blood glucose levels leads to formation of advanced glycosylation end products (AGEs) [13]. Periodontitis results when AGEs react with receptors of advanced glycosylation end products (RAGEs), in the periodontal tissues. Furthermore, animal studies suggest that DM not only induces higher alveolar bone resorption but also alters orthodontic tooth movement [14, 15]. In addition, studies suggest that DM inhibits bone remodeling around the teeth undergoing orthodontic tooth movement [16, 17]. However, to date, no systematic review has been published summarizing and critically analyzing the studies conducted. Therefore, the aim of this review is to systematically summarize the literature concerning the influence of DM on periodontal bone during application of orthodontic forces.

### Materials and methods

#### Focused question

Using the Participants, Intervention, Control and Outcomes protocol described in the Preferred Reporting Items for Systematic Reviews and Meta-Analyses (PRISMA) statement [18], the following research question was constructed: “What is the influence of DM mellitus on periodontal bone during orthodontic tooth movement in diabetic subjects?”

#### Selection criteria

The following types of studies were included in this review: (1) prospective clinical trials, (2) animal studies, (3) studies assessing effect of orthodontic tooth movement in diabetic subjects, (4) tooth movement via coil springs and wires, and (5) studies in English. Reviews, case reports and series, commentaries, letters to the editor, and short communications were excluded.

#### Search methodology

An electronic search was conducted via PubMed/Medline, using the Medical Subject Headings (MeSH) terms “bone remodeling,” “diabetes mellitus,” “orthodontics” and “tooth movement” for studies published between January 2000 and August 2016 by two authors, SN and FS, independently. Similar search was conducted via Google Scholar, Embase, ISI Web of Knowledge, and Cochrane Central Register of Controlled Trials (CONTROL). Any disagreements were solved by discussion. A secondary search was conducted by reading the reference lists of the articles meeting the inclusion criteria for additional studies relevant to this review. A summary for the search criteria and MeSH terms used for searching via PubMed is presented in Table 1.

**Table 1:** A summary of MeSH terms, inclusion criteria, and exclusion criteria used for extracting literature from PubMed for this study

MeSH terms	Inclusion criteria	Exclusion criteria
“Bone remodeling,” “diabetes mellitus,” “orthodontics,” and “tooth movement”	• Prospective clinical trials • Animal studies • Studies assessing effect of orthodontic tooth movement in diabetic subjects • Studies in English	• Reviews • Case reports and series • Commentaries • Letters to the editor • Short communications

*MeSH* Medical Subject Headings

#### Quality assessment of studies

The quality of methodologies employed in the studies was assessed by means of the Animal Research: Reporting of In Vivo Experiments (ARRIVE) guideline [19]^1617^. As shown in Table 2, various aspects of the title, abstract, methodology, results, and discussion were analyzed and assigned quality scores to give an overall score out of 20 in each study.

**Table 2:** General characteristics and outcome of animal studies conducted on the effect of DM on periodontal tissues during orthodontic movement

Study	Animal model (*n*)	Study groups (*n*)	Duration of study	Orthodontic appliance; force magnitude and duration	Outcomes	Reference
Li et al. 2010	48 Sprague–Dawley rats	Group 1: DM + OTM (24) Group 2: healthy + OTM (24)	14 days	0.5 N closed-coil between the incisors and molar applied for 14 days	DM induced higher alveolar bone resorption during OTM	[17]
Braga et al. 2011	60 rats	Group 1: DM + OTM (25) Group 2: healthy + OTM (25) Group 3: DM + OTM + insulin (10)	12 days	35 g Ni–Ti coil spring between the incisors and molar for 12 days	DM induced higher alveolar bone resorption leading to increased OTM	[16]
Villarino et al. 2011	24 Wistar rats	Group 1: DM +OTM (8) Group 2: healthy + OTM (8) Group 3: DM + OTM + insulin (8)	44 days	120 ± 15 g closed-coil spring between the upper molars for 1 week	DM decreased bone remodeling during OTM. Insulin prevented effects of DM on the alveolar bone	[20]
Zhang et al. 2011	48 Sprague–Dawley rats	Group 1: DM + OTM (24) Group 2: healthy + DM (24)	10 weeks	50 g coil spring between the maxillary incisor and molars for 14 days	DM induced higher alveolar bone resorption during OTM	[21]
Plut et al. 2015	24 Wistar rats (healthy) and 24 Goto-Kakizak rats (DM)	Group 1: healthy Wistar + OTM (8) Group 2: Goto-Kakizak + OTM (8) Group 3: healthy Wistar + OTM (8) Group 4: Goto-Kakizak + OTM	42 days	0.25 N coil spring between the left maxillary and second and first molars for entire duration of study	DM reduced bone remodeling during OTM	[22]
Arita et al. 2016	23 Spra- gue-Dawley rats	Group 1: healthy + OTM (7) Group 2: diabetic + OTM (9) Group 3: diabetic + OTM + insulin (7)	28 days	10 g closed-coil spring between palatal miniscrew and the maxillary molar for 2 weeks	DM reduced rate of OTM and decreased bone resorption. Insulin reduced adverse effects of DM on OTM	[15]

*DM* diabetes mellitus, *LLLT* low-level laser therapy, *OTM* orthodontic tooth movement, *TIMP* tissue inhibitor of metalloproteinase

### Results

#### Search results

**Fig. 1:**
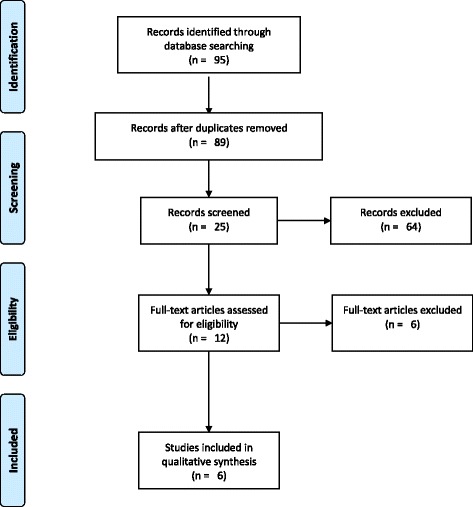
PRISMA flow diagram for the search process employed for this review

After elimination of duplicate items, the primary search resulted in 89 articles. After exclusion of irrelevant articles on the basis of abstract and title, full texts of 25 articles were read to exclude additional irrelevant studies. Six animal studies were included in this review for qualitative analysis [15–17, 20–22]. The PRISMA flow chart (Fig. 1) illustrates the search methodology and results.

#### General characteristics of selected studies

All six studies investigated the impact of DM on periodontal tissues during orthodontic tooth movement [15–17, 20–22]. All studies used rats with experimental DM [15–17, 20–22]. The number of rats used was 23 to 60 [15–17, 20–22]. Duration of studies ranged from 12 days to 2 months, 19 days [15–17, 20–22]. Duration of orthodontic force applied to the teeth ranged from a week to 42 days [15–17, 20–22]. Orthodontic force was expressed in Newton by two studies in which 0.25 and 0.5 N closed-coil springs were placed [17, 22]. In five studies, in which orthodontic force was expressed in grams, 10–120 g of orthodontic force was applied using closed-coil springs [15, 16, 20, 21]. In six studies, closed-coil springs were placed between the maxillary incisors and molars [16, 17, 20–22], while in one study, the spring was placed between the maxillary incisors and miniscrews inserted in the palate [15]. The influence of insulin on DM-affected alveolar bone was investigated in two studies [15, 20]. The general characteristics of the studies are present in Table 2.

#### Main outcomes of selected studies

When compared to healthy animals, more bone resorption and diminished bone remodeling were observed in diabetic animals in all studies [15–17, 20–22]. Furthermore, DM decreased rate of OTM in one study [15], but in another study, DM accelerated OTM [16]. Insulin diminished bone resorption in two studies [15, 20].

#### Results of quality assessment

As shown in Table 3, the quality score of studies ranged from 13 to 17. Majority of the studies did not include appropriate sizes of experiment groups. Additionally, only one study mentioned blinded investigators and operators [17]. In none of the studies, randomization protocols were described. Also, none of the studies stated if any animals had been lost during the duration of the experiments. However, all studied described appropriate statistical analysis and measurement of outcomes. Majority of the studies were carried out for appropriate duration of time. Moreover, only two studies included an appropriate sample size [16, 22].

**Table 3:** Results of the quality assessment of included studies

Study characteristics	Li et al. 2010 [17]	Braga et al. 2011 [16]	Villarino et al. 2011 [20]	Zhang et al. 2011 [21]	Plut et al. 2015 [22]	Arita et al. 2016 [15]
Title	1	1	1	1	1	1
Abstract	1	1	1	1	1	1
Introduction						
Adequate background	1	1	1	1	1	1
Objectives described adequately	1	1	1	1	1	1
Method						
Ethical statement	1	1	1	0	0	1
Blinding	1	0	0	0	0	0
Description of animal groups	1	1	1	1	1	1
Adequate experimental procedures	1	1	1	1	1	1
Experimental animals	1	1	1	1	1	1
Housing	1	0	0	0	0	1
Appropriate sample size	0	1	0	0	0	0
Randomization of animals	0	0	0	0	0	0
Experimental outcomes	1	1	1	1	1	1
Statistics	1	1	1	1	1	1
Results						1
Baseline data	1	1	1	1	1	1
Number analyzed	1	1	1	1	1	1
Adequate outcomes	1	1	1	1	1	1
Reporting of adverse effects	0	0	0	0	0	0
Discussion						
Adequate interpretation of results	1	1	1	1	1	1
Clinical implications	1	1	1	1	1	1
Funding information	1	1	0	0	0	1
Total score (out of 20)	17	16	14	13	13	16

### Discussion

DM has a number of implication on human health. If uncontrolled, it may damage various organs and tissues. Ketoacidosis is a potentially life-threatening condition that may be caused by uncontrolled type 1 DM. Xyrostomia, oral candidiasis, and glossopyrosis are some of the oral diseases commonly observed in patients with uncontrolled DM [23–25]. Moreover, uncontrolled DM may also lead to delayed wound healing and recurrent oral ulceration. DM has also been associated with periodontal disease. Studies indicate that DM increases the risk of periodontal disease by as much as three times when compared in healthy patients [26]. It has been reported that an increased levels of inflammatory biomarkers such as tumor necrosis factor-α (TNF-α), interleukin-6 (IL-6), and c-reactive protein (CRP) are elevated in diabetic individuals and may induce periodontitis [27, 28] It has also been suggested that advanced glycation end products (AGEs), which are produced during DM, interact with receptors of advanced glycation end products (RAGEs) in the periodontal tissues to cause oxidative damage and worsen periodontal disease [29, 30]. Indeed, studies on rodents suggest that periodontitis induced by diabetes diminishes bone formation by favoring apoptosis of osteoblasts and osteoclastogenesis [31].

The altered bone remodeling observed in the studies reviewed in this study [15–17, 20–22] may have been caused by the diminished bone formation observed in prior studies [31]. Application of orthodontic forces leads to creation of strains in the periodontal ligaments (PDL) and alveolar bone around the root of the tooth. This strain leads to the creation of pressure and tension sides in the periodontium tissues surrounding the tooth. Bone resorption on the pressure side and bone formation on the tension leads to bone remodeling which in turn causes tooth movement [32]. Studies suggest that DM not only induces a decrease in osteoclast numbers but also diminishes differentiation of osteoblasts leading to reduced bone remodeling [20]. However, in the study by Plut et al. [22], increased expression of the pro-osteoclastic factor receptor activator of nuclear factor ҡB ligand (RANKL) suggests that DM favors bone resorption by primarily increasing the proliferation of osteoclasts. Indeed, alterations in inflammatory markers such as collagen type I (col-I), metalloproteinase 1 (MMP-1), and tissue inhibitor of metalloproteinase 1 (TIMP1) suggest that DM may increase inflammatory processes in the periodontium leading to more bone resorption [21]. In summary, studies indicate that DM alters bone modeling in periodontium surrounding the tooth. However, more studies are required to ascertain the exact mechanism and the long-term implications of uncontrolled DM on periodontal tissues during application of orthodontic forces.

To date, only two studies have investigated the effect of DM on the rate of orthodontic tooth movement [15, 16]. In the study by Braga et al., accelerated orthodontic tooth movement was detected in diabetic mice [16]. A higher osteoclast count suggested that DM induces orthodontic tooth movement by induced proliferation of osteoclasts. However, observations by Arita et al. [15] indicate that DM reduces resorption of the bone leading to a reduced rate of orthodontic tooth movement. However, this study did not observe the effect of DM on bone cells or periodontal biomarkers; so, the exact reason for diminished orthodontic tooth movement remains unclear. Nonetheless, it is worthwhile to mention that in the studies by Arita et al. and Villarino et al. [15, 20], administration of insulin minimized the effects of DM and increased the rate of orthodontic tooth movement to that of non-diabetic mice, which suggests that if diabetic patients have their blood glucose levels controlled, orthodontic tooth movement will be insignificantly affected by DM. However, more studies are warranted to investigate the effect of insulin on orthodontic tooth movement in diabetic subjects.

The included studies had a number of limitations which have led to biased outcomes. Quality assessment of the included study revealed that there were numerous sources of biased results. None of the studies described blinding of investigators and operators, mentioned randomization of animals, included a predetermined statistically calculated sample size, and carried out method error analysis. Moreover, none of the studies mentioned if any animals were lost during the experiments. Furthermore, more than half the number of the studies did not include appropriate numbers of animals in experimental and control groups [15, 17, 20, 22]. Future research should focus on improving the study design to minimize the potential sources of bias. Another shortcoming among the studies is the varying magnitudes of orthodontic forces employed by each one of them. A lack of standardized methodology may have also contributed to variable results among studies.

Studies suggest that uncontrolled DM may affect the outcomes of orthodontic treatment by altering bone remodeling. A thorough understanding of the various processes, by which uncontrolled DM may affect orthodontic treatment, is necessary. However, the evidence regarding implications of DM on orthodontic tooth movement and bone remodeling is limited to animal studies. To date, no clinical studies have been conducted to assess the effect of uncontrolled DM on the outcomes of orthodontic tooth movement. Nonetheless, orthodontic treatment of patients with uncontrolled DM should be delayed or halted until the disease has been controlled. Orthodontic appliances may lead to easier accumulation of plaque which may worsen existing periodontal disease [33]. Since experimental studies suggest that diabetes may alter bone remodeling [15–17, 20–22], poor oral hygiene may have a synergistic effect on the detrimental effect of the disease on periodontal tissues. Hence, good oral hygiene and periodontal maintenance is imperative for diabetic patients receiving orthodontic treatment.

## Conclusions

DM may adversely affect bone remodeling and tooth movement during application of orthodontic forces. However, more long-term and well-designed studies are required before the exact mechanism and impact of DM on outcomes of orthodontic treatment is understood.
